# Persistent donor derived Vδ4 T cell clones may improve survival for recurrent T cell acute lymphoblastic leukemia after HSCT and DLI

**DOI:** 10.18632/oncotarget.10260

**Published:** 2016-06-23

**Authors:** Ling Xu, Jianyu Weng, Xin Huang, Chengwu Zeng, Shaohua Chen, Suxia Geng, Lijian Yang, Suijing Wu, Suming Huang, Xin Du, Yangqiu Li

**Affiliations:** ^1^ Institute of Hematology, Jinan University, Guangzhou, China; ^2^ Department of Hematology, Guangdong General Hospital, Guangdong Academy of Medical Sciences, Guangzhou, China; ^3^ Key Laboratory for Regenerative Medicine of Ministry of Education, Jinan University, Guangzhou, China; ^4^ Department of Biochemistry and Molecular Biology, University of Florida, Gainesville, Florida, USA; ^5^ Department of Hematology, First Affiliated Hospital, Jinan University, Guangzhou, China

**Keywords:** T-ALL, T cell repertoire, allo-HSCT, MRD, DLI, Immunology and Microbiology Section, Immune response, Immunity

## Abstract

The outcome for T-cell acute lymphoblastic leukemia (T-ALL) in relapse after hematopoietic stem cell transplantation (HSCT) is quite poor, while, both donor lymphocytes infusion (DLI) and adoptively infusion of γδ T cells in leukemia patients after HSCT have demonstrated good results in prolonging survival time of patients. Here, we reported a T-ALL case who experienced three relapses and received HSCT and DLI with an overall survival (OS) time lasting for more than seven years. Based on our previous identification of a leukemic and reactive clone in this patient, continual γδ T cell repertoire monitoring affirmed that the same Vδ5 leukemic clone existed in most samples from the patient, particularly including a sample taken at the time of the third T-ALL relapse, while it could not be detected in the donor sample. In addition, an identical Vδ4 monoclonal T cell that proliferated in the recipient for several years was confirmed to come from the donor graft, and its expression level significantly increased in third leukemia recurrence. These results indicate that clonally expanded Vδ4 T cells may represent a reconstituted γδ T cell repertoire after HSCT, which also hints to a relatively better outcome for this case. Based on this case study, we recommend DLI should be as a treatment strategy for patients who achieve CR or relapse from HSCT. Moreover, dynamically monitoring the TCR repertoire in patients who receive HSCT will benefit in supervising of malignant clone evolution and residue, identifying T cell clones mediate anti-infection, GvHD or GvL.

## INTRODUCTION

T-ALL in relapse is regarded as a practically incurable disease. Although a second completed remission (CR) may be achieved, the median disease-free survival (DFS) for this disease is dismal, but HSCT and DLI may be alternative methods for improving the survival of patients with relapse [1–3]. Here, we report a rare case who experienced three relapses with an overall survival lasting greater than seven years.

In a previous study, we reported a 25-year-old male patient diagnosed with T-ALL on March 2008 who achieved hematological completed remission (CR1) after one course of chemotherapy followed by four courses of consolidation chemotherapy. In November 2009, the patient presented at the Department of Hematology, Guangdong General Hospital and was diagnosed with T-ALL relapse (RE1).

**Figure 1:**
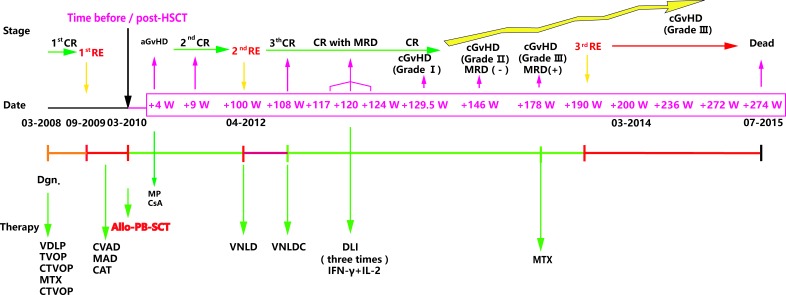
Disease time course of the reported T-ALL case The key dates: initial diagnosis, first time of relapse (RE1), allo-HSCT, RE2 and RE3 are depicted in the middle of the graph. The disease stages: remission or relapse with or without GvHD are shown above the graph, and the type of administered therapy is presented below the graph. Notes: VDLP (V: vineristine, D: daunorubicin, L: L-asparaginase, P: prednisone), TVOP (T: Pirarubicin/THP, V: etoposide, O: vincristine, P: prednisone); CTVOP(C: cyclophosphamide and TVOP ); MTX: methotrexate; CVAD (C: cyclophosphamide, V: vincristine, A: adramycin and D: dexamethasone); MAD (M: Methotrexate, A: Ara-c/cytarabine, D: dexamethasone); CAT (C: cyclophosphamide, A: Ara-c/cytarabine, T: topotecan), VNLD (V: Vindesine, N: mitoxantrone, L: L-asparaginase, D: dexamethasone), VNLDC (VNLD and C: cyclophosphamide); Allo-PB-SCT: allogeneic peripheral blood stem cell transplantation; CR: complete remission; MRD: minimal residual disease.

After being admitted in December 2009 with a recurrent T-ALL diagnosis, the patient received three courses of salvage chemotherapy lasting for three months (specific therapy protocols are shown in Figure 1); however, his response assessments were partial remission (PR), minor remission (MR), and MR respectively. Considering the poor prognosis for relapse T-ALL [2], the patient received an HLA-identical sibling peripheral blood (PB) HSCT on March 2010. Fifteen days after HSCT, a second complete remission was achieved (CR2). Short tandem repeat (STR) analysis revealed a 100% donor chimera. Four weeks after HSCT, the patient suffered grade IV skin acute GvHD (aGvHD), which was ultimately controlled with methylprednisolone (MP) and cyclosporin (CsA) treatment at eight weeks post-HSCT [4]. Central nervous system leukemia (CNSL) was found 20 weeks post-HSCT; thus, intrathecal chemotherapy and radiotherapy of the head were then applied [5]. The patient continued CR2 and remained normal upon cerebrospinal fluid examination at 40, 52, and 68 weeks post-HSCT. However, relapse (RE2) was detected again 100 weeks after HSCT, approximately 33.5% lymphoblasts revealed in bone marrow (BM). After receiving chemotherapy over the next two months, the patient achieved his third remission (CR3; 108 weeks post HSCT) with minimal residual disease (MRD) detected by flow cytometry (FCM).

Except the standard clinical and laboratory examination, to characterize the leukemia T cell clone, we used the fine-tiling comparative genomic hybridization (FT-CGH), ligation-mediated polymerase chain reaction (LM-PCR), real-time PCR (RT-PCR), GeneScan and sequencing techniques and identified the T-ALL clone, which included a monoclonally expanded TCR Vδ subfamily member with a Vδ5-Dδ2-Jδ1 rearrangement [6]. This molecular characteristic provide a useful maker for monitoring the prognosis of this case. We also simultaneously identified a clonally expanded reactive donor-derived Vδ4 T cell clone (rearranged as Vδ4-Dδ3-Jδ1) that existed in all samples from the recipient post-HSCT, which may function as an anti-leukemia T cell clone that may improve patient survival.

In this study, we continually monitored the TCR γδ repertoire in this patient from 117 weeks post-HSCT until the patient died at 274 weeks post-HSCT due the third relapse (RE3/the second relapse post-HSCT).

Based on careful analysis and dynamically monitoring the TCR γδ repertoire in this patient, we tried to sum up our experience with recurrent T-ALL therapy by evaluating information from dynamic TCR γδ repertoire monitoring.

## RESULTS

### Treatment after HSCT and disease progression

For the purpose of eradicating MRD, instead of receiving adjuvant chemotherapy, the patient received 3 donor lymphocyte infusions (DLIs) at 117, 120 and 124 weeks post-HSCT (Figure 1), and IFN-γ and IL-2 were given to enhance the DLI effects. At week 129.5 post HSCT, the patient underwent mild cGvHD (Grade I of mouth), so IL-2 was ceased, and the frequency of IFN-γ was adjusted to once per month. At week 146 post-HSCT, the mild cGvHD progressed to moderate cGvHD (Grade II with skin). At week 178 post HSCT, the patient suffered severe cGvHD (Grade III of skin, sclerosis-like), FCM analysis revealed MRD with 0.14% blast cells, while 2% blast cells were detected in the BM by a marrow aspirate smear (BMAS). Thus, we ceased the IFN-γ therapy and used methotrexate (MTX) (10 mg q.w.) for cGvHD treatment. At week 190 post-HSCT, the patient was diagnosed with T-ALL relapse (RE3) with BMAS demonstrating 8% lymphoblast. At 200-week post-HSCT, lymphoblast were increased to 90% in BM and 14% in PB. However, what was interesting is that the severe cGvHD continued to exist even after the patient underwent an obvious relapse. Finally, the patient refused to receive further chemotherapy and died at 274 weeks post-HSCT (July 28, 2015). The treatment used and efficacy are summarized in Figure 1 and the MRD monitoring FCM figure is provided as Supplemental Figure 1.

### TCR γδ repertoire monitoring of leukemic and donor-derived T cell clones

The patient achieved an extended disease-free survival time from CR3 to the time before the third leukemia recurrence, which lasted 82 weeks. We continually monitored the dynamic changes in the TCR Vδ5 subfamily in this case at different time points from CR3 to the endpoint before the patient died (117, 120, 135, 142, 146, 178, 190, 200, 236 and 272 weeks post-HSCT respectively), and the TCR γ and δ repertoires of the donor samples were also analyzed at the same time. Sample collection dates and disease statuses are shown in Table 1.

**Table 1:** Sample collection dates and clinical patient characteristics

Collection Date	Diagnosis	Smear analysis Blast cells (%) In BM / PB	MRD analyzed by FCM	Disease Statuses
31.08.2012	Donor			
14.08.2012	117W post allo-HSCT	3 / 0	2.55%	CR, before DLI
31.08.2012	120W post allo-HSCT	3 / 0	6.36%	CR, before DLI
19.12.2012	135W post allo-HSCT	3 / 0	NA	CR, Grade I cGvHD
05.02.2013	142W post allo-HSCT	2 / 0	NA	CR, Grade I cGvHD
05.03.2013	146W post allo-HSCT	2 / 0	NA	CR, Grade II cGvHD
16.10.2013	178W post allo-HSCT	2 / 0	0.14%	CR, Grade II cGvHD
14.01.2014	190W post allo-HSCT	8 / 1	3.81%	Relapse, Grade III cGvHD
25.03.2014	200W post allo-HSCT	90 / 14	87.78%	Relapse, Grade III cGvHD
14.10.2014	236W post allo-HSCT	NA	NA	Relapse, Grade III cGvHD
14.07.2015	272W post allo-HSCT	96/97	96.67%	Relapse, Grade III cGvHD
27.07.2015	274W post allo-HSCT			Died

Notes: NA: Not analyzed, CR: complete remission, MRD: minimal residual disease, DLI: donor lymphocyte infusion, cGVHD: chronic graft versus host disease.

**Figure 2:**
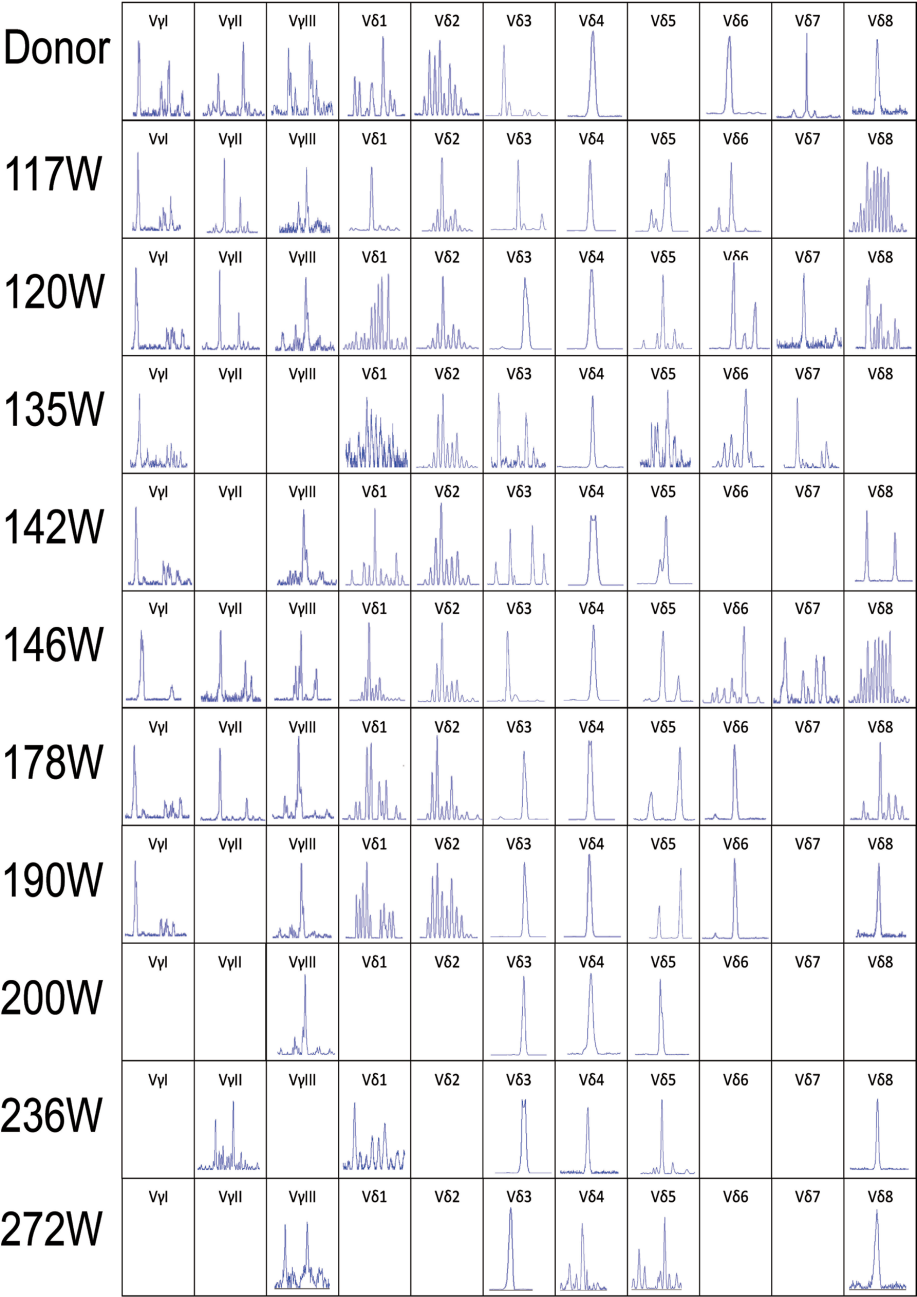
CDR3 spectratyping of the TCR Vγ and Vδ subfamily T cells in peripheral blood from the donor and recipient (117 to 272 weeks post-HSCT) at different time points after allo-HSCT The 466bp Vδ5 clone was not detected in the donor sample and the recipient sample in 178 weeks post-HSCT, the same size Vδ4 monoclone could be detected in the donor and recipient samples in all time points post-HSCT. All the Vδ3, Vδ4 and Vδ5 clones detected in the same size are shown in the same position in horizontal axis. CDR3, complementarity determining region 3.

**Figure 3:**
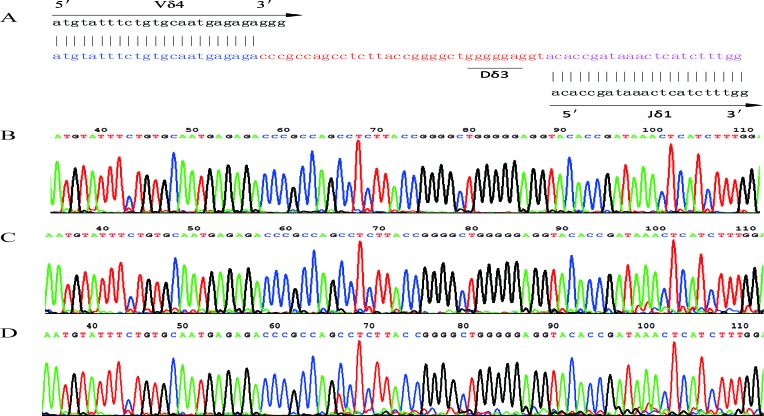
The nucleotide sequence of the Vδ4 CDR3 region **A.** The sequence of the Vδ4 CDR3 region in the donor that rearranged as Vδ4-Dδ3-Jδ1. **B.** The sequence of Vδ4 CDR3 region of recipient at 4 weeks post-HSCT. **C.** The sequence of Vδ4 CDR3 region in recipient at 120 weeks post-HSCT. **D.** The sequence of Vδ4 CDR3 region in recipient at 190 weeks post-HSCT. CDR3, complementarity determining region 3.

**Figure 4:**
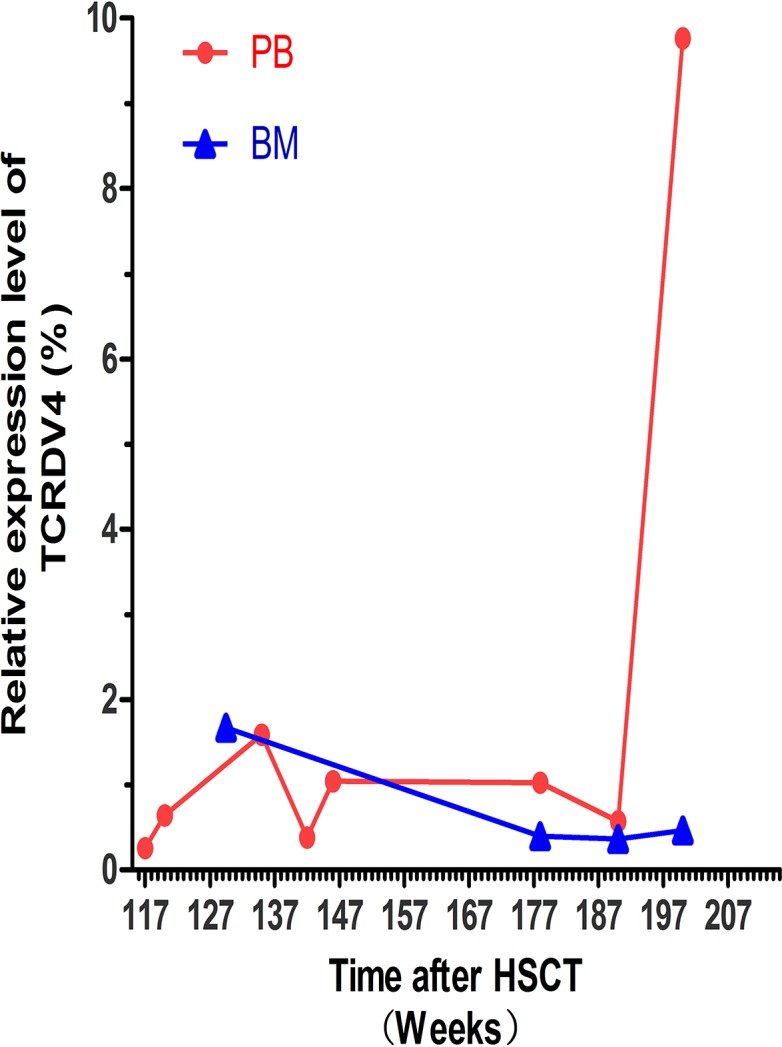
The TCR Vδ4 expression level in the recipient at different time point post-HSCT The red dots indicate the expression level of the TCR Vδ4 (TCDV4) gene in PBMCs at the 117 W, 120 W, 135 W, 142 W, 146 W, 178 W, 190 W and 200 W time point post-HSCT. The blue triangles show the expression level of the TCR Vδ4 genes in the bone marrow (BM) at the 129.5 W, 178 W, 190 W and 200 W time point post-HSCT.

**Figure 5:**
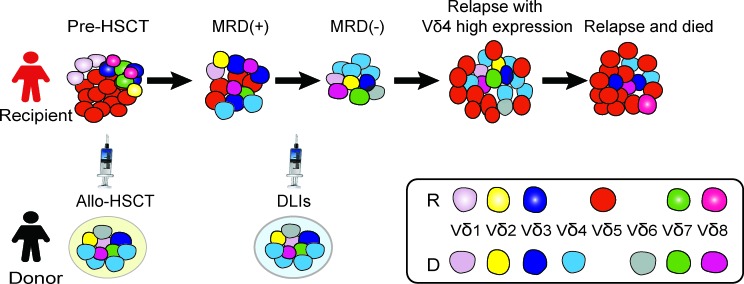
A schematic diagram of γδ T cell and leukemic clone evolution in the case with T-ALL The red cells represent Vδ5 leukemic clone identified in this case and not exist in the donor sample, this leukemic clone nearly undetectable when the patient achieved CR with 0.14% MRD detected by FCM, while it appeared again when the third time of disease relapse; the sky-blue cells represent Vδ4 monoclone T cell detected in the donor sample and the same Vδ4 T cell clone expanded in the recipient samples post-HSCT; the cells fill with gradient dark blue represent 526 bp Vδ3 T cell clone detected in the patient sample pre-HSCT (RE1) and several samples after-HSCT, especially in the recipient samples collected in RE3, but not exist in the donor sample, while, cells fill with dark blue (without gradient) represent the 508 bp clonally expanded Vδ3 T cells identified in the donor sample which also can be detected as a monoclone in the recipient samples in 117,135 and 142 weeks post-HSCT.

The leukemic Vδ5 T cell clone, which we previously identified at the time of diagnosis, could be detected in all of the recipient samples with exception for one collected at 178 weeks post-HSCT when the patient was in CR status (Figures 1 and 2) with 0.14% blast cells in the BM (Table 1). As expected, this Vδ5 leukemic clone could not be detected in the donor sample (Figure 2), indicating that this oligoclonal Vδ5 clone (with same CDR3 length, PCR products: 466 bp) did not come from the donor, and it appeared with disease relapse and disappeared or decreased with disease remission. The oligoclonally expanded Vδ4 subfamily T cell clone (Vδ4-Dδ3-Jδ1; Figure 3B), which was detected in all samples post-HSCT (4, 8, 68, 100, and 108 weeks), but not in the sample before HSCT [1], was also detectable in all samples in this study with the exception of the sample (272 weeks post-HSCT) collected before the patient died (Figure 2). Direct sequencing confirmed that all of the CDR3s of the oligoclonally expanded Vδ4+ subfamily rearranged in the same pattern and were consistent with those previously detected (Figure 3). Interestingly, the same monoclonal Vδ4 clone was also identified in donor sample collected at the time for DLI. The CDR3 sequence of the Vδ4 clone in donor sample was confirmed to be consistent with that in the recipient (Figure 3A). In addition, the Vδ4 expression level was significantly increased to a new peak at the time of RE3 (200 weeks post-HSCT), and it was undetectable in the next two samples (236 and 272 weeks post-HSCT). The Vδ4 expression level in the PBMC and BMC samples at different time points are shown in Figure 4. Moreover, the dynamic changes in the TCR Vδ3 subfamily attracted our attention, clonally expanded Vδ3 T cells with a 508 bp (PCR products) was identified in the donor sample can be also detected a monoclonal expansion in the recipient samples in 117,135 and 142 weeks post-HSCT, while another Vδ3 T cell clone with a 526 bp (PCR products) was detected in the patient sample pre-HSCT (RE1) and several samples after-HSCT, but not exist in the donor sample, and had been identified in the same sequence (Figure 2). The evolution of Vδ3 T cell clone depicted in Figure 5 and Supplemental Figure 2. The evolution of γδ T cell clones from both donor and recipient are summarized in a schematic figure (Figure 5).

## DISCUSSION

The disease process of this T-ALL case from the time of initial diagnosis to the final relapse can be divided into three phases: initial diagnosis to CR1 and then undergoing RE1 (Phase 1), treatment with allo-HSCT to achieving CR2 and then undergoing RE2 (Phase 2), treatment with salvage chemotherapy and achieving CR3 followed by death due to RE3 (Phase 3). It is well known that leukemic relapse in adult ALL remains a major therapeutic challenge. The outcome for recurrent T-ALL is quite dismal with only 5% having a 5 year overall survival (OS) [7]. For the patient in this study, the common chemotherapy used allowed him to achieve remission. Unfortunately, disease recurrence could not be controlled with salvage chemotherapy. As several clinical trials have demonstrated, for patients who have not already been treated with HSCT, the achievement of a second remission is usually followed by high-dose chemotherapy and HSCT because it is typically regarded as the only potentially curative therapeutic option and the main goal of treatment after relapse [7–10]. However, the patient relapsed at 100 weeks post-HSCT (RE2), VDLP (vineristine, daunorubicin, L-asparaginase and prednisone) regimen help the patient achieved CR3, but MRD could be detected, which was thought to be a risk for relapse; therefore, DLIs were used to reduce the leukemic cells. Significantly, the MRD was controlled by this useful adoptive immunotherapy, and the patient successfully remained in CR3 for 20 months before the leukemia relapsed again at 190 weeks (RE3) post-HSCT. These results indicate that using DLI to eradicate residual leukemic clones in T-ALL patient after HSCT is quiet useful and helpful for prolonging the survival of patients and preventing leukemia recurrence. Allen et al. reported similar results in a study in which DLI successfully controlled MRD and led to a disease-free survival of more than 3 years for a 9-year-old T-ALL patient [11]. These results indicate that DLI may be used as a common strategy for T-ALL patients with MRD after HSCT. Even after undergoing RE3, the OS time of the patient was much longer than expected. The long-term disease remission of this patient and serial DLI treatment after achieving a third CR with persistent cGvHD were signals reflecting that an immune response was mediated by the grafts or the infusion of cells from the donor [12, 13]. However, as we knew, there were also examples that DLI was failed to rescure patients with relapse leukemia after HSCT. The mechanism of the DLI which could successfully control the malignant leukemia clone in this case is worthy to characterize. In this study, we will discuss the clinical status combined with the immune effects detected by dynamic TCR repertoire monitoring in the following section in the hope that this will deepen our understanding of the underlying immunological mechanisms involved in this process.

The identification of leukemic T cell clones at the time of diagnosis is important for evaluating disease remission, MRD, relapse and the possibility of clonal evolution in T cell malignancies. There are few studies reported the distribution of different Vδ subfamily leukemia, in previous studies, we identified two T-ALL cases with Vδ1 and Vδ2 bi-malignant T cell clones [14]. Based on the leukemic clone we identified at the initial time of disease and by monitoring the TCR repertoire in this case, the monoclonally expanded TCR Vδ5 subfamily with the Vδ5-Dδ2-Jδ1 rearrangement sequence was considered to be a component of the leukemic cell in this case together with the TCR VγI subfamily. This stable Vδ5 T cell clone indicated that this patient developed γδ T cell leukemia, which is rare compared with αβ T cell leukemia, representing approximately 10% of T-ALL cases. Moreover, even for γδ T-ALL, most leukemic T cells are largely expressed with limited Vδ1-Jδ1 rearrangements as reported by Langerak et al. who showed only 6 of 30 γδ T-ALL expressed non-Vδ1 [15]. Comparison of the non-Vδ1+ TCR γδ T-ALLs with the more common Vδ1+ type revealed a heterogeneous mixture with respect to the immunophenotypic markers (e.g., CD1, CD4 and CD8, which are often expressed by non-Vδ1+ TCR γδ T-ALLs). In addition, a higher frequency of complete TCR δ and TCR β rearrangements and ‘end-stage’ Jg2.3 gene rearrangements are often detected in non-Vδ1+ TCR γδ T-ALLs, and these suggest a non-Vδ1 TCR γδ T-ALL trend toward more mature immunophenotypes compared with Vδ1+ types. The immunophenotypic markers we reported for this Vδ5+ T-ALL case did not include CD1, CD4 or CD8. However, this patient expressed CD13, which is a marker that might suggest higher relapse risk compared with CD13^−^ T-ALL cases [9]. Further analysis of TCRβ and ‘end-stage’ Jg2.3 gene rearrangements in the leukemic clone may help clarify the deterioration stage of this T-ALL patient. In addition, comparing the surface markers expressed between Vδ1+ T-ALLs and non-Vδ1+ TCR γδ T-ALLs may provide useful information for predicting the prognosis of these patients. In this study, the Vδ5 leukemic clone represented when disease relapsed, we did not determine the leukemic clone evolution with other Vδ subfamily by monitoring TCR repertoire based on clonal expansion status. Obvious, most Vδ subfamily T cells could be detected in most samples at the time point from 117 to 190 weeks, however, polyclonal expansion or oligoclonally expanded T cells with different CDR3 lengths indicated that came from different clones and would be the unspecific expansion. This T cell expansion may be enhanced by IL-2 and IFN-γ which were used with DLI [16, 17]. However, even the relapse with the same Vδ5 leukemic clone, whether there was evolution in another molecule that may affect the biological behavior of a leukemic clone remains unclear, this may be identified by deep sequencing of the patient samples, which were collected at each instance of disease recurrence [18–20].

The DLI treatment had a positive effect on controlling the minor leukemic clone in this case, which prompted our interest in whether there was any anti-leukemia clone proliferation in the recipient after HSCT. Previously, we detected a monoclonally expanded Vδ4 T cell clone in the patient samples after, but not before HSCT. Interestingly, we detected this same clone not only in the recipient samples but also in the donor sample, indicating that this T cell clone expanded in the donor for unknown reasons. It may be possible that a memory T cell subset could respond to a virus infection and encounter the same viral antigen in the recipient body after HSCT; however, there was a decrease and ultimate loss of this Vδ4 monoclone together with disease recurrence and death. We also suspected that this clonally expanded T cell may mediate an anti-leukemia effect in the recipient, and it may have a cross response to the epitopes expressed in the leukemic T cells in this case. Increasing data have shown that γδ T cells play a critical role in anti-tumor function. Lamb and colleagues found that the five year leukemia-free survival (LFS) and OS of acute leukemia patients with increased γδ T cells after HSCT was significantly higher compared with those with normal/decreased γδ T cells. Meanwhile, this group also demonstrated *in vitro* that original donor γδ T cells are less likely to mediated GvHD effects [21–23]. Most recently, Vδ1 and Vδ2 T cell clones expanded *in vivo* have been demonstrated to efficiently lyse primary lymphoid and myeloid blasts in patients who received HLA-haploidentical transplantation (haplo-HSCT) [24–27]. These results may support that the donor derived Vδ4 T cell clone we identified here may be specific to any T-ALL antigen and work efficiently for lysing T-ALL blasts. For confirming the function of Vδ4 T cells, TCR γδ gene could be cloned from this Vδ4 T cells and transferred to healthy T cells to detect the specific cytotoxicity [28, 29]. If the anti-leukemia effect could be confirmed, such clonally expanded Vδ4 T cells in this case may be considered as specific DLI to avoid GvHD development. In addition, we observed that the Vδ4 expression level in the PBMC significantly increased, while no in the bone marrow, the reason may be due to the difference of T cell numbers, the homing of donor T cells and the different microenvironment, it may be interesting to farther investigation. However, even oligoclonal Vδ3 T cells were identified, it seemed more complex to address. Because, the clonally expanded Vδ3 T cells with 508 bp identified in both donor and recipient samples obviously originated from the donor, and it is possible that possesses a specific anti-leukemia function or alternative functions. While the Vδ3 T cell clone with 526 bp detected only in the patient sample pre-HSCT (RE1) and several samples after-HSCT might be a minor leukemic clone in this case, because it appeared at the time of pre-HSCT and MRD but was more obvious and monoclonal when the patient underwent RE2 and RE3. However, the function of the clonally expanded Vδ3 requires further confirmation.

In conclusion, we reported a T-ALL case who experienced three relapses and received HSCT and DLI with an OS time lasting for more than seven years, persistent clonally expanded Vδ4 T cells may hint to a relatively better outcome for this case. However, the genetic or epigenetic alteration as well as the tumor microenvironment may be the important factors to influence the outcome and survival [30, 31], for example, low expression of T-cell transcription factor BCL11b predicts inferior survival in adult standard risk T-ALL patients, our previous study showed overexpression of *BCL11b* in T-ALL samples including sample from this case [32], is this one of the possible role in this case with unusually long survival, it remains an open question. Although this T-ALL case lived much longer than most relapse T-ALL patients, disease recurrence still indicates a worse final outcome even for cases of late relapse (relapses after 17 years have been reported), and their prognosis after relapse may be poor as reported [33–35]. Therefore, it is important to maintain the first complete remission as long as possible. In addition, γδ T cell repertoire as well as TCRαβ analysis is an important tool for monitoring malignant T cell clone and detecting MRD, while donor derived γδ T cells might have potential GVL effects without significant GvHD effects. Recently, a new subtype of memory T cells, stem cell memory T (T_SCM_) cells, was shown to be associated with superior T cell engraftment, persistence, and anti-tumor immunity in HSCT. Monitoring human T_SCM_ cells may help to evaluate the immune status for such patients [36, 37].

## MATERIALS AND METHODS

### Sample preparation

The patient and donor peripheral blood (PB) samples were collected in EDTA-containing collection tubes, and PB mononuclear cells (PBMCs) were separated using the Ficoll-Hypaque gradient centrifugation method. RNA was extracted using TRIzol (Invitrogen, Carlsbad, CA, USA), and it was then reverse-transcribed into first-strand cDNA for TCR repertoire amplification using random hexamer primers and the High Capacity cDNA Reverse Transcription kit (ABI, Carlsbad, CA, USA) according to the manufacturer's instructions. This study was approved by the Ethics Committee of the Medical School of JINAN University of Guangdong Province in China, and all procedures were conducted according to the guidelines of the Medical Ethics Committees of the health bureau of the Guangdong Province of China.

### RT-PCR for TCR Vγ and Vδ subfamily member amplification

Three sense TCR Vγ primers and a single Cγ reverse primer, or eight TCR Vδ sense primers and a single Cδ primer were used in an unlabeled PCR to amplify the TCR Vγ and Vδ subfamilies, respectively. PCR was performed as described in our previous report [38]. Aliquots of the cDNA (1 μl) were amplified in 20 μl with 1 of the 3 Vγ primers with a Cγ primer or 1 of the 8 Vδ primers with a Cδ primer. The final mixture contained 0.5 μM sense and antisense primer, 0.1 mM dNTP, 1.5 mM MgCl_2_, 1× PCR buffer and 1.25 U Taq polymerase (Promega, USA). The amplification was performed in a DNA thermal cycler (BioMetra, Germany) with a 3 min denaturation at 94°C and 40 PCR cycles. Each cycle consisted of 94°C for 1 min, 60°C for 1 min and 72°C for 1 min with a final 7 min elongation at 72°C. All PCR products were analyzed in a 1.5% agarose gel stained with ethidium bromide and then stored at 4°C prior to GeneScan analysis.

### GeneScan analysis for TCR Vγ and Vδ subfamily clonality

Positively expressed and unlabeled PCR products (3 μl) were subjected to a cycle of a runoff reaction with a fluorophore-labeled Cγ-FAM or Cδ-FAM primer. The labeled runoff PCR products (2 μl) were heat denatured at 94°C for 4 min with 9.5 μl formamide (Hi-Di Formamide, ABI, USA) and 0.5 μl Size Standards (GenenScan-500-LIZ™ PerkinElmer, ABI). The samples were then loaded in a 310 POP-4™ gel (Performance Optimized Polymer-4, ABI, USA) and resolved by electrophoresis with a 310 DNA sequencer (ABI, Perkin Elmer) for size and fluorescence intensity analysis using GeneScan software [39–41].

## SUPPLEMENTARY MATERIAL AND FIGURES


