# Supplementary material for: Persistent donor derived Vδ4 T cell clones may improve survival for recurrent T cell acute lymphoblastic leukemia after HSCT and DLI
Source: Oncotarget. 2016 Jun 23;7(28):42943–52. doi: 10.18632/oncotarget.10260 (PMC5189998; doi:10.18632/oncotarget.10260)
Supplement: Supplementary file 1 [file oncotarget-07-42943-s001.pdf]

Persistent donor derived Vδ4 T cell clones may improve survival for recurrent T cell acute lymphoblastic leukemia after HSCT and DLI

Supplementary Material

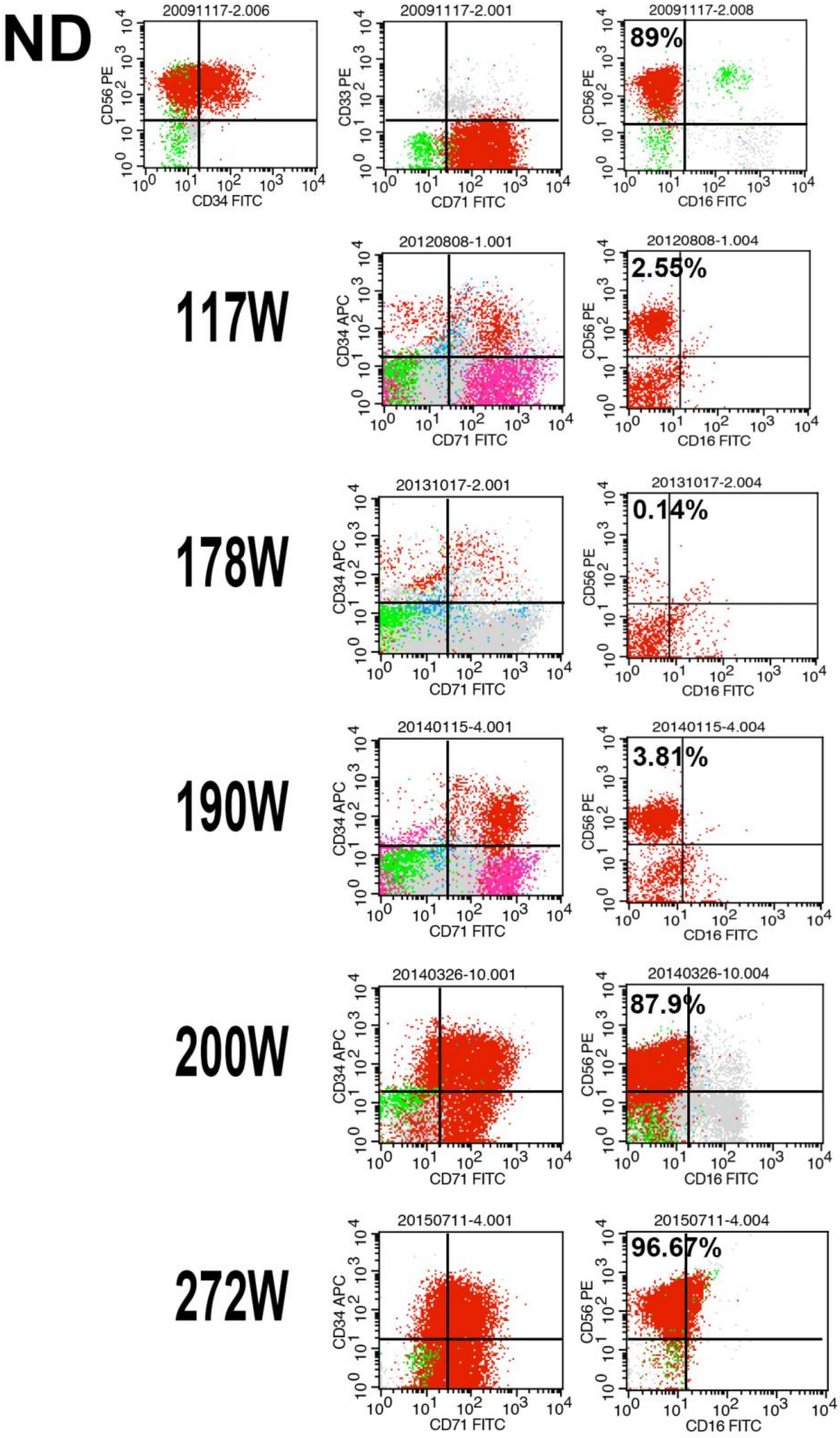

Supplemental Figure S1 : Flow cytometry (FCM) monitoring MRD of the T-ALL patient at different time points. CD34, CD71, CD16, CD56 were used as individually MRD monitoring marker of this patient. The percentage of lymphoblasts at various disease states (ND, 117 W, 178W, 190W, 200W and 272W) were shown in the 2D plot figure (CD65 PE and CD16 FITC). Red scatter points represent the lymphoblast in bone marrow. Pink, green, and gray dots represent nucleated erythrocytes, mature lymphocytes and mature granulocytes, respectively, which gated in 2D spot figure (CD45-Percp and Side Scatter-Height).ND, newly diagnose; MRD, minimal residual disease.

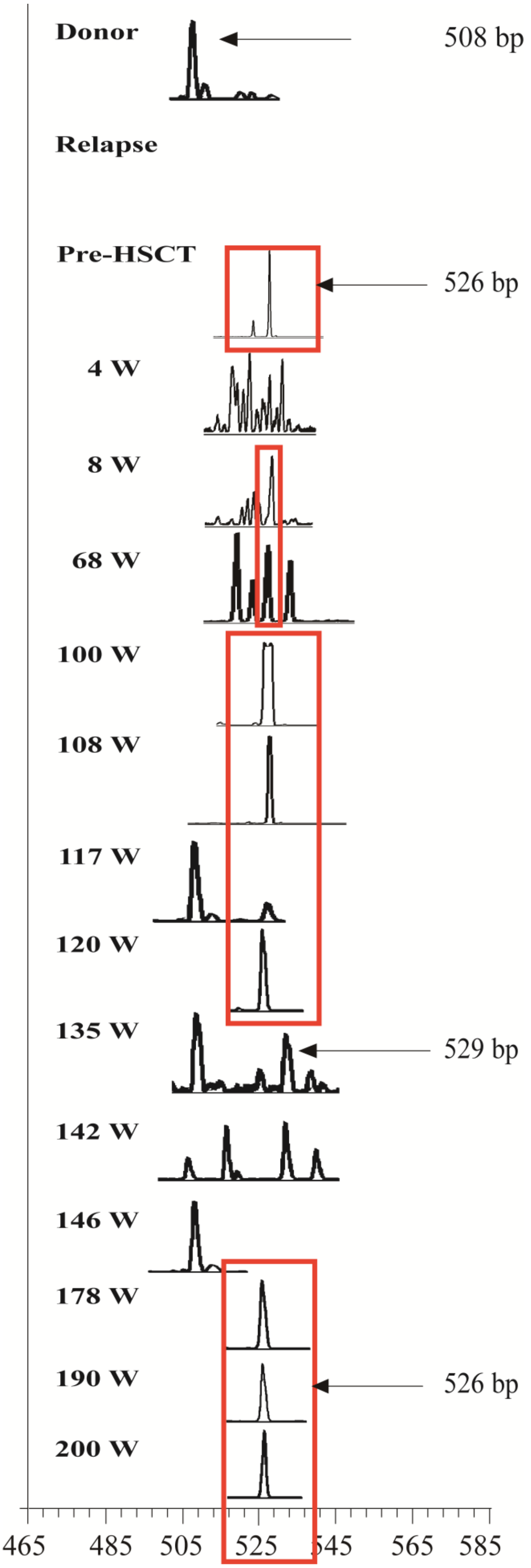
Donor
508 bp
Relapse
Pre-HSCT
526 bp
4 W
8 W
68 W
100 W
108 W
117 W
120 W
135 W
529 bp
142 W
146 W
178 W
190 W
526 bp
200 W
465
485
505
525
545
565
585

Supplemental Figure S2 : The CDR3 spectratyping of Vδ3 T cells in the samples of donor and recipient at different time points post-HSCT. The red boxes indicated the clonally expanded Vδ3 T cell clone with 526 bp which contained the same sequence confirmed by PCR product direct nucleotide sequencing. The 508 bp clonal expanded Vδ3 T cell detected in the donor also detected in the recipient samples in 117, 135 and 146 weeks post-HSCT. CDR3, complementarity determining region 3.
